# Repositioning community-based mental health care for people with psychosocial disabilities: a physical health equity–oriented whole-health recovery framework

**DOI:** 10.3389/fpsyt.2026.1993750

**Published:** 2026-10-22

**Authors:** Yan Zhang, Yunhua Cao

**Affiliations:** Department of Physical Education, North China Electric Power University, Beijing, China

**Keywords:** community-based rehabilitation, physical activity, physical health equity, psychosocial disability, severe mental illness, social prescribing, whole-health recovery

## Abstract

Community-based mental health care has expanded identification, treatment, continuity of care, and rehabilitation for people with psychosocial disabilities, yet physical health, functional capacity, and social participation remain insufficiently integrated into routine support. This Perspective proposes a physical health equity-oriented whole-health recovery framework that builds on existing community mental health services. The framework follows a single service logic in which routine contact identifies physical, functional, and participation needs; needs and support assessment considers clinical status, functional limitations, social barriers, preferences, and available resources; and needs-matched support facilitates participation, adaptation, and continued engagement. Its distinctive proposition is that disproportionate loss among people facing greater health or participation barriers should trigger further assessment, adaptation, or support with identifiable service responsibility. Equity is therefore examined across identification, assessment, matching, participation, retention, and benefit. Supported community participation provides one means of translating identified needs into everyday community life, with social prescribing representing one possible implementation mechanism. The proposed sequence is intended as a testable service proposition rather than a validated universal pathway, and its implementation should be examined across different service and policy contexts.

## 1 Introduction

Community mental health care has expanded access to identification, assessment, treatment, follow-up, crisis response, and rehabilitation. Global reforms increasingly emphasize person-centered, rights-based, and recovery-oriented services that enable people to live in their communities (1–3). Recovery should therefore include not only clinical improvement, but also the meaningful opportunities to participate in everyday community life (4, 5).

Physical health remains a critical yet insufficiently integrated dimension of that agenda. People with severe mental illness (SMI) experience a high burden of physical multimorbidity, cardiovascular disease, diabetes-related complications, and premature mortality (6–9). The Lancet Psychiatry Commission consequently framed physical health protection as a core responsibility of mental health systems (10). Yet monitoring and treatment recommendations do not, by themselves, explain how community-based mental health care can be extended so that physical health equity becomes a routine component of recovery.

Physical health equity adds a distributional question to this clinical agenda. It asks not only whether assessment or intervention exists, but also who can access and receive appropriate physical health care, whether support is responsive to different levels of need, and who remains engaged long enough to benefit (11). Equal availability may therefore coexist with unequal use and unequal outcomes. People with greater symptom burden, poverty, mobility limitations, stigma, or weak social support may face the highest physical health risks while being least able to act on standard advice. A service-system framework must therefore make these gradients visible and connect them to responsibility, resource allocation, and review.

Terminology requires care. This Perspective uses psychosocial disability as a rights- and participation-oriented concept that emphasizes the interaction between mental health conditions and social barriers. However, most epidemiologic evidence on mortality, cardiometabolic risk, and exercise interventions comes from people diagnosed with schizophrenia-spectrum disorders, bipolar disorder, major depressive disorder, or populations grouped under the category of SMI. Accordingly, although we adopt psychosocial disability as the broader conceptual lens, the empirical evidence discussed in this Perspective largely concerns people with severe mental health conditions. The framework should not be interpreted as assuming uniform clinical risks or service needs across all people with psychosocial disabilities. Throughout this Perspective, empirical claims are therefore attributed to the diagnostic populations from which the evidence is drawn, rather than generalized to the broader category of psychosocial disability.

This Perspective proposes a physical-health-equity-oriented whole-health recovery framework. Its central claim is that community care can build on its existing mental health functions by making physical health, functional capacity, and supported participation explicit governance targets. Here, repositioning does not imply replacing or devaluing the established functions of community-based mental health care. Here, repositioning does not imply replacing or devaluing the established functions of community-based mental health care. It refers to broadening its health horizon so that physical health, functional capacity, and social participation become routine components of recovery-oriented support. Within this broader approach, exercise social prescribing is considered one practical means of linking identified needs with supported participation in community settings.

The framework draws on several established strands of work. Whole-health and integrated-care scholarship has broadened attention beyond psychiatric symptoms and emphasized coordination across mental, physical, and social care (12–15). Community-based rehabilitation offers a complementary perspective by placing functioning, empowerment, inclusion, and participation within everyday community life at the center of support for people with disabilities, as reflected in the WHO Community-Based Rehabilitation Guidelines (16). Social prescribing further provides a practical means of connecting people with non-clinical resources and supportive relationships in their communities (17–19). Building on this work, the present framework focuses on what happens as people move through the service process. It asks whether those facing greater physical-health needs or participation barriers are identified, receive support that fits their circumstances, remain engaged, and ultimately benefit. Physical health equity is therefore used to examine where unequal loss occurs across identification, assessment, matching, participation, retention, and benefit, and where service responsibility may need to be strengthened.

RE-AIM offers a well-established framework for examining reach, effectiveness, adoption, implementation, and maintenance at program and population levels (20). The present framework focuses more specifically on transitions within community mental health care. It asks where people facing greater physical-health or participation barriers are disproportionately lost as they move through identification, assessment, support matching, participation, follow-up, and benefit. When such unequal loss occurs, it is treated as a signal for further assessment, adaptation, or support, with responsibility assigned to an identifiable service actor. This proposition can be examined through differences in referral completion, retention, adaptation, and benefit across groups. RE-AIM may subsequently be used to evaluate the implementation of such a service model, while the present framework specifies the service sequence and response rule being evaluated. With this distinction established, the sections that follow examine where physical-health needs may lose visibility within routine community care and how those transitions can be made more responsive and accountable.

## 2 Why physical health remains insufficiently integrated into community-based mental health care

Community mental health care necessarily prioritizes symptoms, medication, relapse, crisis, and continuity. Physical health, function, and participation often extend across longer time horizons and organizational boundaries, which makes them easier to leave outside routine care.

First, continuity of mental health care does not equal good health. A person may remain in contact with services while developing medication-related weight gain, hypertension, diabetes, pain, poor sleep, low fitness, or frailty. Longstanding monitoring gaps and fragmented responsibilities remain documented concerns (11, 21). Second, physical health sits between specialist mental health care and primary care. Recent reviews show that integrated models can improve access and preventive screening, but effects on clinical outcomes are mixed and implementation depends on coordination, information systems, workforce capacity, and sustained leadership (12, 13). Third, an emphasis on behavior advice can misclassify constrained opportunity as weak motivation. People may face fatigue, poverty, unsafe environments, stigma, low confidence, limited transport, or uncertainty about appropriate activity. Fourth, family members often become the default coordinators of medication, daily routines, appointments, and crises. This can make preventive care and participation feel secondary. Fifth, digital systems can strengthen continuity, but a check-in or risk flag does not necessarily help someone interpret health information, reach a primary care visit, or enter a welcoming community activity.

This mismatch is reinforced by the way routine work is recorded. Contact, medication adherence, relapse, and crisis events are relatively easy to define, count, and assign. Function, pain, fatigue, sleep, confidence, mobility, and social participation are more dispersed across clinical and everyday settings. They may be documented inconsistently or treated as matters outside the mental health team’s remit. What is measured then shapes what is discussed in supervision, financed by commissioners, and recognized as successful practice. Physical health inequity can therefore persist without deliberate neglect. It emerges when responsibilities are fragmented, outcomes are weakly specified, and no actor is accountable for helping a person move from risk identification to sustained participation.

The distinction between recommendation and capability is especially important. Advice often assumes that a person has the resources, confidence, practical support, and a current health condition that allows them to respond safely. Supported participation treats these conditions as part of the intervention. It locates capability within relationships and environments rather than individual motivation alone, directing attention toward modifiable service barriers.

## 3 A physical health-equity-oriented whole-health recovery framework

The proposed physical-health-equity-oriented whole-health recovery framework treats physical health as an integral condition of recovery rather than an additional service domain. It builds on the established functions of community-based mental health care and broadens their field of vision to physical health, functional capacity, everyday activity, agency, and social participation. From an equity perspective, the central issue is not simply whether a service exists, but whether people with different levels of need and different barriers can be identified, supported, remain engaged, and benefit. Whole-health recovery therefore refers to a multidimensional process in which mental health recovery, physical health, function, agency, and community inclusion are supported within a connected community care environment.

This repositioning involves three interconnected extensions of community-based mental health care. First, the outcome horizon is broadened to include physical health, functional capacity, agency, and social participation within recovery. Second, routine support is extended beyond clinical follow-up and general behavioral advice to include the identification of physical and functional needs, needs-matched support, and sustained community participation. Third, evaluation is widened beyond service contacts and completion indicators to examine function, participation, access, retention, and the distribution of benefits across population groups. These extensions do not constitute separate pathways; together, they describe a single broadening of what community-based mental health care recognizes, supports, and evaluates. Recent whole-health and recovery scholarship similarly argues that biological, psychological, and social domains must be integrated rather than treated as parallel service silos (14, 15).

The framework follows a single service logic. Routine community contact creates an opportunity to identify physical health, functional, and participation needs that may otherwise remain weakly integrated into mental health care. Needs and support assessment then considers clinical safety, functional limitations, social barriers, personal preferences, and available resources. Matching converts these needs into feasible forms of support, while accompaniment and community coordination help people move from recommendation to actual participation. Continued feedback allows support to be adapted as health status, preferences, symptoms, or living circumstances change. When participation becomes sustained and meaningful, it can contribute to physical health, function, confidence, social connection, and valued community roles. Equity is assessed across this entire process by asking who is reached, who remains engaged, who benefits, and where people facing greater barriers are lost.

Across this process, support should remain proportionate to need, continuous across service transitions, respectful of individual agency, and accountable for unequal access, retention, and benefit. **Figure 1** summarizes this single service logic as a cyclical process in which equity-oriented evaluation informs continued identification, adaptation, and support. Together, these principles distinguish whole-health recovery from a checklist of screening tasks or a narrow exercise promotion program.

**Figure 1:**
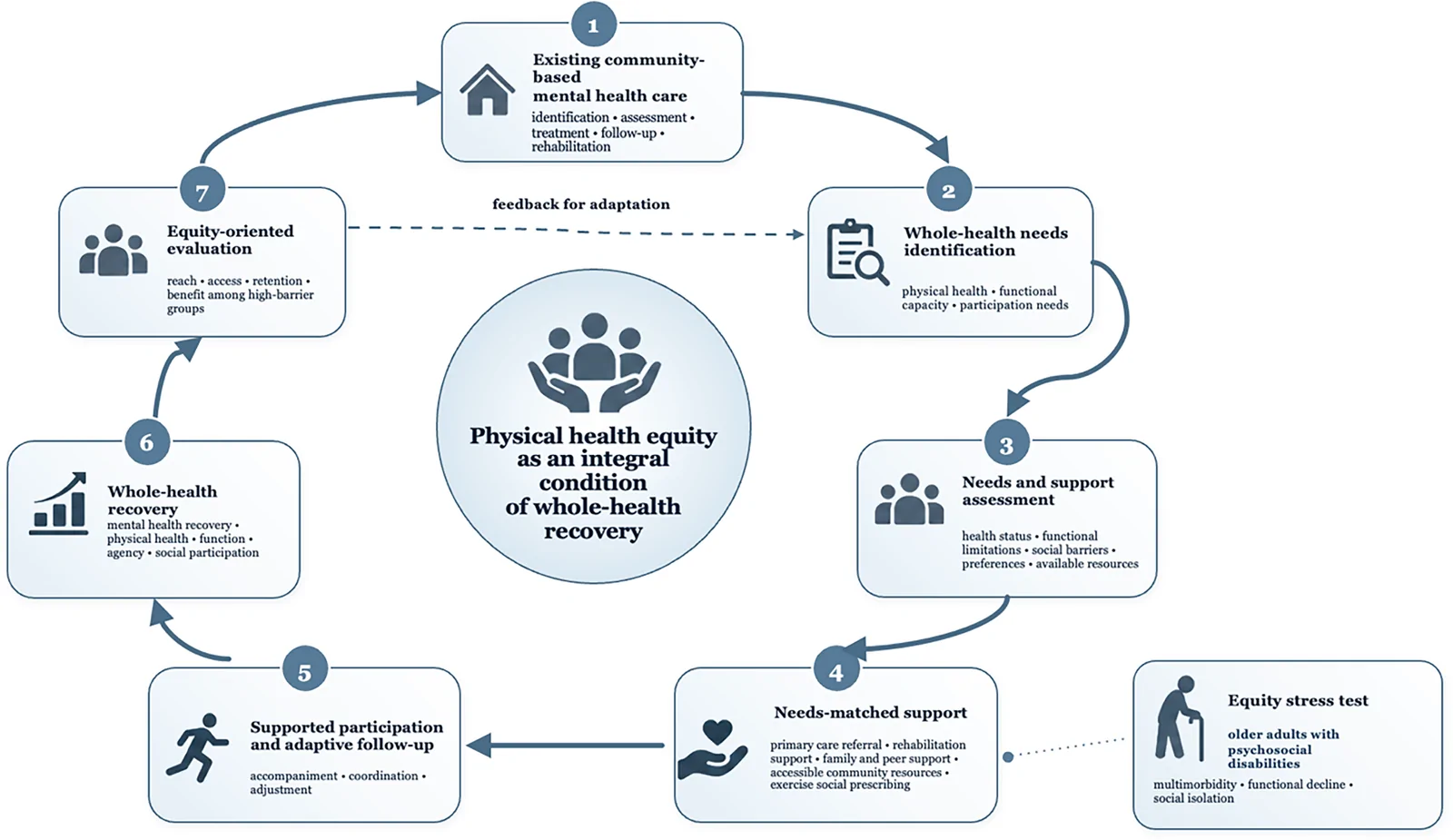
A physical health-equity-oriented whole-health recovery framework.

**Figure 1** integrates these elements into a single cyclical framework. Existing community-based mental health care provides the institutional starting point, while routine community contact creates opportunities to identify whole-health needs, assess the level and form of support required, and connect people with needs-matched resources. Supported participation and adaptive follow-up then help translate available services into sustained engagement in everyday community life. Whole-health recovery is treated as a multidimensional outcome encompassing mental health recovery, physical health, function, agency, and social participation. Equity-oriented evaluation closes the loop by examining who is reached, who remains engaged, who benefits, and where people facing greater barriers are lost, with these findings feeding back into subsequent identification, matching, and adaptation. The framework therefore treats physical health equity not as a separate intervention target, but as an integral condition of whole-health recovery across the community care process.

## 4 Supported community participation: translating the framework into practice

Within this broader framework, supported community participation provides the practical bridge between identified whole-health needs and everyday community life. Physical activity is useful because it can affect fitness, symptoms, daily function, confidence, and social contact. However, low activity among people with schizophrenia, bipolar disorder, and major depressive disorder is well established, and intervention effects vary by outcome and delivery conditions (22, 23). A 2025 meta-review found benefits across several mental, cognitive, physical, and functional outcomes, while also emphasizing implementation barriers and the absence of evidence for physical activity as a universal first-line treatment (24). Evidence for metabolic outcomes remains less certain (25). These findings support activity as one component of care, not as a substitute for medical treatment or structural reform.

Exercise social prescribing is used here as one illustrative form of supported community participation rather than as the defining intervention of the framework. Its value lies less in the act of referral itself than in connecting assessment, matching, accompaniment, adaptation, and sustained participation. Social prescribing research suggests that outcomes depend on person-centered relationships, practical support, community capacity, and continued engagement rather than referral alone (17–19). For people facing frailty, multimorbidity, isolation, digital exclusion, or environmental barriers, a weekly exercise target is insufficient unless services also protect function and participation (26). Supported participation need not be limited to exercise. Arts-based and other community activities may also support social connection, meaning, and participation when they are accessible and matched to individual needs and preferences (27).

The implementation sequence has six linked stages. Whole-health identification brings physical health, function, everyday activity, and participation needs into routine community contact. Needs and support assessment considers health status, functional limitations, social barriers, personal preferences, and available resources. Support matching connects identified needs with feasible responses, which may include primary care referral, rehabilitation, exercise social prescribing, peer or family support, and accessible community resources. Supported participation then helps translate referral or advice into actual engagement through accompaniment, coordination, and adaptation. Adaptive follow-up treats nonparticipation or discontinuation as information about fit, safety, burden, or context and adjusts support accordingly. Community inclusion and role development create opportunities for sustained participation to develop into meaningful relationships and valued social roles. Together, these stages specify a proposed pathway through which routine community contact could be connected with whole-health recovery.

Each stage also has an equity-sensitive failure mode. Identification without follow-through produces documentation rather than support. Needs assessment fails when greater needs become grounds for exclusion rather than greater adaptation. Support matching fails when only a narrow range of options is available. Supported participation becomes fragile when accompaniment depends entirely on unpaid relatives or inaccessible services. Follow-up loses value when discontinuation is treated as non-adherence rather than information about mismatch, burden, or changing circumstances. Inclusion remains superficial when attendance does not develop into voice, relationships, or valued roles. Equity therefore needs to be examined across transitions, because disadvantage can accumulate through small losses at multiple points in the service sequence.

Clinical urgency takes precedence over participation support. New or worsening physical symptoms, clinically significant test abnormalities, serious medication adverse effects, acute mental deterioration, or self-harm and suicide risk require timely clinical assessment and escalation before, or in parallel with, community participation planning. Medication-related metabolic effects, sedation, pain, and mobility limitations may require psychiatric or primary-care review rather than an activity prescription alone (28, 29). Once urgent or clinically significant needs have been addressed, functional and participation barriers can be considered through rehabilitation, community support, or social prescribing (30).

Safety and autonomy should then be addressed together Participation planning should identify contraindications and adaptation needs, with procedures for falls, heat, pain, distress, and referral to primary or specialist care when required. Participants should be able to choose among activities, set personally meaningful goals, and withdraw without jeopardizing access to other services. Supported participation therefore complements appropriate clinical care rather than substituting for it.

In China, walking groups, Tai Chi, Qigong, chair-based movement, and other locally available activities may provide feasible options when matched to individual function and preference, but no single activity defines the proposed pathway (23).

## 5 Extending community-based mental health care in China

China’s community mental health system provides a substantial platform for this extension. The 686 Project helped move care for people with severe mental illness beyond an exclusively hospital-based model by strengthening continuity between specialist services, primary care, and community follow-up. Its service model incorporated assessment, access to medication, regular follow-up, referral, rehabilitation guidance, health education, and support for patients and families, creating an infrastructure for continuing treatment and community-based rehabilitation (31, 32). Subsequent reforms further embedded these functions within community and primary-care services, with recent studies documenting continued development in treatment continuity, follow-up coverage, and community service provision (33, 34). This existing infrastructure therefore provides a practical starting point for extending routine community care toward physical health, functional capacity, and participation as components of whole-health recovery.

China’s National Basic Public Health Service Specification already provides a formal basis for physical-health attention among registered people with severe mental disorders. It requires regular follow-up and, when clinically feasible and consented to, an annual health examination that includes general physical examination and relevant laboratory and cardiovascular assessments (35). Routine follow-up also includes attention to physical illness, medication adverse effects, functioning, medication use, and relevant examination results. The gap addressed by the present framework is therefore not the absence of formal physical-health monitoring. It concerns what happens after a need or abnormality becomes visible: whether clinically significant findings receive appropriate review or referral, whether referrals are completed, whether functional limitations lead to rehabilitation or adaptation, and whether people facing greater barriers remain connected to care and community resources. However, these possible losses should not be treated as an implementation failure. Incomplete examination, weak follow-through, referral failure, limited rehabilitation access, and unequal retention are distinct problems that may vary across settings. The framework makes these transition points explicit so that their relative importance can be examined empirically. The national program also applies to a defined population of registered people with severe mental disorders and should not be treated as coterminous with the broader category of psychosocial disability. The broader framework may therefore involve additional entry points through mental health care, primary care, disability services, and community organizations.

Recent evidence also shows substantial improvement in community management while documenting regional variation in service indicators and incomplete use of community rehabilitation, reinforcing the need to examine how service pathways operate across settings (34). Rural community data document physical comorbidity among people with SMI, while a large Jiangsu study found substantial excess life-years lost and cause-specific mortality (9, 36). Peer support and community-based rehabilitation studies show growing attention to social functioning, quality of life, family burden, and implementation conditions (37–39). These developments create a foundation for linking mental health care with primary care, disability services, community organizations, and local physical activity resources.

Operational responsibility should be assigned task by task, not vaguely described as shared. Mental health teams retain responsibility for psychiatric assessment, medication review, symptom change, acute deterioration, and clinical escalation. Primary care leads physical-health assessment, management of common chronic conditions, and referral when abnormalities require further investigation or treatment. Rehabilitation, community, and disability services can address functional barriers and organize accessible opportunities for participation. Activity professionals adapt intensity and safety where relevant. Peer supporters and family members may accompany participation by choice, but should not become unpaid substitutes for professional responsibilities.

This division of labor requires an identifiable coordinating function. Coordination could sit with a community mental health worker, primary care team member, rehabilitation practitioner, or trained link worker, depending on local capacity. The essential feature is not a new professional title, but ownership of the transition between sectors. Where no existing role has the capacity to perform this function, coordination should be treated as an additional service input rather than assumed to occur within existing workloads. The coordinator should confirm that assessment leads to an agreed plan, that referrals are completed, and that feedback returns to the relevant clinical and community teams. Without this function, intersectoral collaboration can remain an aspiration while people and families continue to navigate disconnected services alone.

Governance should also align information and financing with the pathway. A minimum shared record could include physical health needs, functional limitations, activity preferences, agreed adaptations, referral status, adverse events, and reasons for discontinuation. Data sharing must remain proportionate, consent-based, and protected from use as generalized surveillance. Financing should recognize coordination, accompaniment, adaptation, and community capacity building as service inputs rather than treating only clinical contacts or completed sessions as productive activity. Local review can then identify where people leave the pathway and whether losses are concentrated by rurality, income, age, disability, or diagnosis. Tasks already contained within existing clinical and follow-up responsibilities should remain within those services. Additional work generated by cross-sector coordination, repeated accompaniment, adaptation, and pathway tracking requires protected staff time, commissioned partnerships, or additional funding rather than being absorbed invisibly into routine workloads. Its value can be examined through completed referrals, retention, functional change, patient-reported benefit, adverse events, and the distribution of these outcomes across population groups.

The framework also distinguishes components that can be introduced through near-term workflow changes from those that require additional system capacity. Within current community mental health structures, routine contacts could add brief identification of physical-health, functional, and participation needs; primary-care referral and follow-up could be made more systematic; existing disability and neighborhood services could map accessible community resources; and case review could record referral completion, retention, and reasons for nonparticipation (38). By contrast, dedicated coordination time or link-worker functions, cross-sector training, interoperable or shared records, funded accompaniment and adaptation, and accountability for equity indicators would require explicit workforce, financing, and governance arrangements rather than being absorbed into already stretched routine roles. These system-level enablers are therefore best developed through staged local piloting and investment (31–34, 36, 37, 39).

## 6 Discussion: implications, boundaries, and research priorities

The contribution of this framework is not to introduce whole-health recovery as a new goal, nor to present social prescribing as a universal solution. Its contribution lies in connecting physical health equity to the performance of the entire community care sequence. Equity can be lost during identification, needs assessment, support matching, entry into participation, continued engagement, or follow-up even when services are nominally available. The framework therefore shifts attention from whether services exist or whether average outcomes improve to whether people facing greater barriers can move through the service sequence, remain engaged, and benefit without being disproportionately lost.

Digital tools may support navigation, adaptive follow-up, and communication when they remain embedded within human care relationships. Their contribution to the proposed pathway is prospective and requires population-specific evaluation, particularly where digital access or usability may itself create barriers (40, 41).

The framework has clear boundaries. Psychosocial disability is broader than SMI, while much of the cited physical health evidence comes from diagnostic SMI populations. Social prescribing evidence remains heterogeneous, commonly observational, and sensitive to local capacity. Exercise cannot correct inadequate medical treatment, poverty, inaccessible environments, discrimination, or fragmented financing. The six-stage sequence should therefore be understood as a testable service proposition rather than evidence that implementation will itself improve physical health, participation, or social inclusion. It specifies where support and accountability may need to change; its effects require prospective empirical evaluation.

Older adults provide one useful equity stress test because multimorbidity, functional limitations, sensory impairment, mobility restrictions, and changing social or caregiving circumstances may accumulate within the same person. A pathway that works mainly for people who are already mobile, confident, and socially connected may improve average participation while leaving those with greater barriers behind. Older age therefore offers one context in which the accessibility and adaptability of the proposed service sequence can be examined, without implying a uniform risk profile among older people with psychosocial disabilities (42, 43).

Research should now focus on four priorities. First, studies should measure physical health and functional inequities within community mental health cohorts, with stratification by diagnosis, age, sex, socioeconomic position, rurality, disability, and treatment exposure. Second, pragmatic and hybrid implementation-effectiveness designs should test the full support chain, not activity effects alone. Third, common outcome sets should include access, referral completion, retention, function, participation, adverse events, and distributional equity alongside symptoms and biomarkers. Fourth, governance studies should examine financing, accountability, data sharing, workforce roles, and the sustainability of community assets.

The unit of evaluation should match the claim. Individual outcomes can show changes in fitness, symptoms, function, or quality of life, but cannot establish an equitable system alone. Service evaluation should examine reach, completed transitions, adaptation, and retention. Population evaluation should test whether health and participation gaps narrow across groups. Qualitative work should examine dignity, belonging, cultural fit, and reasons for leaving, while economic evaluation captures coordination and accompaniment costs.

Study designs should also separate intervention efficacy from pathway performance. An activity may benefit attendees while the referral system loses people with the greatest barriers. Hybrid designs can identify whether failure arises from the activity, matching, support, or service environment. Co-design with people with psychosocial disabilities, families, peers, clinicians, and community providers should shape acceptable outcomes and strategies, treating lived experience as knowledge about pathway performance.

The next phase of community mental health reform is not simply to ask people with psychosocial disabilities to exercise more. It is to make physical health, function, and participation visible within routine support, and to build the relationships and local pathways through which whole-health recovery becomes possible.

## Data Availability

The original contributions presented in the study are included in the article/supplementary material. Further inquiries can be directed to the corresponding author.
